# Comparative Performance of the COMHON Index and the Braden Scale for Pressure Injury Risk Assessment in Critically Ill Patients: A Retrospective Observational Study Using Consecutive ICU Sampling

**DOI:** 10.1155/nrp/9154245

**Published:** 2026-11-03

**Authors:** Sandra Mas Serván, Sandra Giao Varela, María Victoria Crespo Torres, Sonsoles Hernández Iglesias, Mercedes Gómez del Pulgar, Miguel Angel Cuevas-Budhart, Johis Ortega, Alina Renghea, Almudena Crespo-Cañizares

**Affiliations:** ^1^ Nursing Department Hospital Universitario Quirónsalud Madrid Madrid Spain; ^2^ Nursing Education Coordination Quirónsalud Private Hospitals Madrid Spain; ^3^ Corporate Nursing Management Grupo Quirónsalud Madrid Spain; ^4^ Department of Nursing Faculty of Health Sciences Universidad Francisco de Vitoria Pozuelo de Alarcón Madrid Spain ufv.es; ^5^ Medical Research Unit in Nephrological Diseases Centro Médico Nacional Siglo XXI Instituto Mexicano del Seguro Social Mexico City Mexico imss.gob.mx; ^6^ School of Nursing and Health Studies University of Miami Coral Gables Florida USA miami.edu; ^7^ Institute of Health and Sport Sciences Faculty of Health Science Universidad Francisco de Vitoria Madrid 28223 Spain ufv.es

## Abstract

**Background:**

Pressure injuries remain a major patient safety concern in critically ill patients admitted to intensive care units (ICUs). Although the Braden Scale is widely used for pressure injury risk assessment, its performance may be limited in highly complex critical care settings. The COMHON Index was developed as an ICU‐specific tool integrating physiological variables related to tissue perfusion and oxygenation.

**Aim:**

To compare the performance of the COMHON Index and the Braden Scale for pressure injury risk assessment in critically ill patients.

**Study Design:**

A retrospective,longitudinal, comparative observational study was conducted in a single ICU inSpain between January 2023 and December 2024, using consecutive nonprobabilitysampling.

**Results:**

A total of 476 critically ill patients were included; ICU‐acquired pressure injuries developed in 1.9% of patients during the first 24 h after ICU admission. Greater disease severitywas associated with a higher risk of pressure injury. Higher total Braden scores showed a protective effect, whereas COMHON domains related to consciousness, mobility, hemodynamic stability, and oxygenation were directly associated with pressure injury development. The COMHON Nutrition subscale did not reach statistical significance in univariable analysis (OR: 1.38; *p* = 0.303). In multivariable analysisusing Firth’s penalized logistic regression, impaired nutritional status(COMHON) was associated with pressure injury, whereas the Braden sensoryperception and moisture exposure subscales showed inverse associations. At the Youden‐optimized cutoffs (Braden ≤ 10; COMHON ≥ 15), the Braden Scale demonstrated 100.0% sensitivity (95% CI: 66.4–100.0) and 77.1% specificity (95% CI: 73.0–80.8), while the COMHON Index demonstrated 88.9% sensitivity (95% CI: 51.8–99.7) and 73.2% specificity (95% CI: 69.0–77.2). Both instruments showed very high negative predictive values (Braden: 100.0%; COMHON: 99.7%).

**Conclusions:**

Both instruments were associated withpressure injury occurrence. The final multivariable model incorporated physiological domains from both instruments, two Braden Scale subscales (sensory perception and moisture exposure) and one COMHON Index domain (nutritional status), suggesting complementary rather than substitutive clinical utility. These findings should be interpreted cautiously given the retrospective single‐center design and low event rate. Prospective multicenter studies are needed to confirm the clinical utility of ICU‐specific risk assessment tools in pressure injury prevention.

**Relevance to Clinical Practice:**

Integrating ICU‐specific physiological parameters into pressure injury risk assessment may support earlier identification of critically ill patients requiring intensified preventive interventions, including repositioning protocols, advanced pressure‐redistributing surfaces, nutritional optimization, and closer hemodynamic monitoring. These findings may contribute to more individualized nursing care planning and improved allocation of preventive resources in intensive care settings.

**Impact Statement:**

This study supports the use of ICU‐specific risk assessment tools to strengthen patient safety strategies and optimize individualized nursing interventions in critical care settings.

**Summary**
• What Is Known About This Topic
◦ Pressure injuries are a frequent and serious patient safety issue in critically ill patients.
◦ The Braden Scale is widely used for pressure injury risk assessment but may have limited performance in intensive care units.
◦ Physiological instability plays a key role in pressure injury development in critical illness.
• What This Paper Adds
◦ Both the COMHON Index and the Braden Scale identified pressure injury risk in critically ill ICU patients, with the final predictive model incorporating subscales from both instruments.
◦ Physiological domains related to perfusion, oxygenation, and nutritional status were strongly associated with early pressure injury risk.
◦ ICU‐specific risk assessment tools may enhance clinical decision‐making and prevention strategies in critical care nursing.

## 1. Introduction

Pressure injuries are localized damage to skin and tissue, usually over bony prominences from sustained pressure or shear [1, 2]. They are a significant safety problem, linked to increased morbidity, longer hospital stays, higher costs, and reduced quality of life [3]. Their development reflects the interplay between mechanical load and tissue tolerance, influenced by perfusion and oxygenation [1].

In intensive care units (ICUs), the incidence of pressure injuries may exceed 20% in some critically ill populations [1, 4]. Critically ill patients are especially vulnerable due to factors such as immobility, altered consciousness, mechanical ventilation, hemodynamic instability, impaired perfusion, systemic inflammation, and use of vasoactive drugs [1]. Therefore, identifying patients at high risk is considered a cornerstone of pressure injury prevention strategies [1, 3]. For this purpose, risk assessment scales have been widely implemented in clinical practice to support decision‐making and to guide preventive nursing interventions. Among these instruments, the Braden Scale has been among the most widely used risk assessment instruments worldwide and has demonstrated acceptable performance in general hospitalized populations [5‐6]. Nevertheless, its generalist design and limited integration of physiological parameters may reduce its sensitivity in critically ill patients, particularly in those with deep sedation, advanced organ dysfunction, or hemodynamic compromise [7‐8].

Recent systematic reviews and validation studies continue to report variability in the predictive accuracy of conventional pressure injury risk scales in intensive care populations, particularly in patients with severe organ dysfunction and prolonged hemodynamic compromise [8‐9]. In response to these limitations, the COMHON Index, comprising level of Consciousness, Mobility, Hemodynamics, Oxygenation, and Nutrition, was developed specifically for use in ICU populations [4, 10]. By incorporating physiological variables directly related to tissue perfusion and oxygen delivery, the COMHON Index was designed to provide a more context‐specific assessment of pressure injury risk in critically ill patients. Subsequent studies have evaluated the COMHON Index in multiple international contexts, including Australian ICU populations [4] and a Chinese Mandarin adaptation demonstrating acceptable inter‐rater reliability and convergent validity with the Braden Scale [10]. Comparative evidence evaluating the clinical performance of the COMHON Index and Braden Scale under real‐world ICU conditions in European settings, however, remains scarce. Furthermore, the relative contribution of physiological instability domains to early pressure injury development during critical illness has not been fully established in this context.

In addition to risk assessment, effective prevention of pressure injuries in critically ill patients requires the systematic implementation of evidence‐based nursing interventions. In this regard, the importance of nutritional status as a determinant of tissue integrity and repair capacity has been highlighted, particularly in patients with prolonged critical illness [11‐12]. Furthermore, the use of structured tools, such as clinical checklists, has been shown to improve adherence to pressure injury prevention and treatment actions in nursing practice [13].

Given the persistently high burden of pressure injuries in critically ill populations and the need for more physiologically informed prevention strategies in intensive care settings, further comparative evaluations of pressure injury risk assessment tools are warranted. Therefore, the aim of the present study was to compare the performance of the COMHON Index and the Braden Scale in assessing pressure injury risk among critically ill patients admitted to an ICU. We hypothesized that the COMHON Index, given its ICU‐specific physiological domains, would demonstrate superior sensitivity for identifying patients at risk for early pressure injury development compared with the Braden Scale in this retrospective cohort.

## 2. Materials and Methods

### 2.1. Study Design and Setting

A single‐center retrospective longitudinal comparative observational study was conducted in the ICU of Hospital Universitario Quirónsalud Madrid, Spain, between January 2023 and December 2024. The study was designed to compare the performance of the Braden Scale and the COMHON Index for pressure injury risk assessment in critically ill patients under routine clinical conditions.

### 2.2. Study Population and Sampling Strategy

The study population consisted of patients aged ≥ 18 years who were consecutively admitted to the ICU between January 2023 and December 2024. Consecutive nonprobability sampling was used, and all patients meeting the eligibility criteria during the study period were included. This approach was selected to minimize selection bias and to reflect a real‐world ICU population.

All ICU admissions were retrospectively identified and systematically screened through electronic clinical records using predefined eligibility criteria. Patients were included if documented pressure injury risk assessment data were available within the first 24 h after ICU admission in the electronic clinical records. Patients presenting with a pressure injury at ICU admission were excluded to restrict the analysis to incident events.

Data extraction was performed using standardized abstraction procedures based on routinely collected electronic clinical records in order to reduce potential information bias.

Given the retrospective observational design, no a priori sample size calculation was performed. The final sample size was determined by the number of eligible patients admitted during the study period, in accordance with standard methodological practice for retrospective cohort studies.

### 2.3. Data Collection

Demographic and clinical data were extracted from electronic health records using standardized data abstraction procedures. Collected variables included age, sex, weight, height, primary diagnosis category, comorbidities including diabetes mellitus, and global severity of illness assessed using the SAPS‐3 score at ICU admission.

Pressure injury risk was assessed using two instruments within the first 24 h of ICU admission:

Braden Scale: Routinely documented by nursing staff in the electronic clinical records as part of standard care and retrospectively extracted for analysis. Both the total score and individual subscale scores were included in the analysis.

COMHON Index: Calculated retrospectively based on documented clinical parameters available within the first 24 h of ICU admission. Thisretrospective approach differed from prospective bedside administration bynursing staff; scores were reconstructed from routinely documented clinical data rather than administered in real time, which represents a methodological limitation discussed further below. The index integrates the level of consciousness, mobility, hemodynamic status, oxygenation, and nutritional status. Each domain is scored on a scale of 1–4, with a total score ranging from 5 to 20. In the published validation, scores ≥ 14 were classified as high risk [4]. In this sample, the Youden index identified ≥15 as the optimal cutoff, slightly higher than the published thresholdand is reported alongside the original validated cutoff in Table 1 for transparency. Individual components and the total COMHON score were analyzed.

**TABLE 1:** Predictive performance of the Braden Scale and the COMHON Index at Youden‐optimized cutoffs.

Parameter	Braden Scale	COMHON Index
Cutoff used	≤ 10 (Youden index)	≥ 15 (Youden index)
Sensitivity (%)	100.0 (66.4–100.0)	88.9 (51.8–99.7)
Specificity (%)	77.1 (73.0–80.8)	73.2 (69.0–77.2)
Positive predictive value (%)	7.8 (3.6–14.2)	6.0 (2.6–11.5)
Negative predictive value (%)	100.0 (99.0–100.0)	99.7 (98.4–100.0)

Sensitivity, specificity, PPV, and NPV with 95% CIs (Clopper–Pearson) for PI occurrence within 24 h (*n* = 476; events *n* = 9). Youden‐optimized cutoffs: Braden ≤ 10; COMHON ≥ 15. For reference, the original published COMHON high‐risk threshold is ≥ 14 [4]. Wide CIs reflect the small event count.

The use of preventive measures, including pressure‐redistributing mattresses, was also recorded.

### 2.4. Outcome Variable

The primary outcome was the development of an ICU‐acquired pressure injury during the first 24 h after ICU admission, as documented in nursing and clinical records. This time window reflects the documentation practices of the study center, where initial risk assessment was systematically recorded within the first 24 h. The implications of this restriction on event rate and representativeness are addressed in the Limitations section.

### 2.5. Study Variables

Continuous variables included age, weight, height, SAPS‐3 score, total Braden Scale score, total COMHON Index score, and individual subscale scores.

Categorical variables included sex, presence of diabetes mellitus, primary diagnosis category, and use of pressure‐redistributing mattresses.

### 2.6. Management of Missing Data

The proportion of missing data was low across most variables, except for height (missing in 17.8% of patients; *n* = 85). To preserve sample size and reduce potential bias associated with complete‐case analysis, missing values were handled using multiple imputation. Two independent imputation methods were applied: k‐nearest neighbors (KNN) and multiple imputation by chained equations (MICE). Both approaches yielded consistent results for variable selection and effect estimates; therefore, the final imputed dataset was used for analysis.

### 2.7. Statistical Analysis

Descriptive statistics were used to characterize the study population. Continuous variables were evaluated for distributional assumptions and are reported as mean ± standard deviation or median with interquartile range, as appropriate. Categorical variables are presented as absolute frequencies and percentages.

Associations between baseline clinical variables and pressure injury occurrence were first examined using univariable logistic regression. Given the low event rate, Firth’s penalized likelihood approach was applied to mitigate small‐sample bias and ensure stable effect estimates [14].

A multivariable logistic regression model was then developed to identify independent predictors of pressure injury development. Variable inclusion was based on clinical relevance and model parsimony, using a stepwise selection strategy informed by the Akaike information criterion. Collinearity among candidate predictors was assessed and found to be noninfluential. The final model included four predictors, yielding an events‐per‐variable ratio of approximately 2.25 (9 events/4 predictors), which falls below the commonly recommended threshold of ≥ 10 [15]. Although Firth’s penalized regression mitigates separation bias, model instability and the potential for unreliable confidence intervals in this setting cannot be excluded. Results from the multivariable analysis should therefore be interpreted as exploratory and hypothesis‐generating.

The use of pressure‐redistributing mattresses was significantly associated with pressure injury occurrence in univariable analysis (OR: 5.12, *p* = 0.012). This variable was excluded from the multivariable model on the basis that its association is likely attributable to reactive prescription driven by nursing risk assessment, representing confounding by indication rather than an independent causal pathway. This reasoning is acknowledged as a study limitation.

Model discrimination was evaluated using receiver operating characteristic curve analysis with 10‐fold cross‐validation repeated 100 times. Discriminative performance was quantified by the area under the curve and corresponding 95% confidence intervals. Given the small number of events (*n* = 9), the AUC estimate should be interpreted with caution as it is likely subject to optimism bias; bootstrap‐corrected or externally validated estimates would be required to confirm this figure. Model calibration was assessed using the Hosmer–Lemeshow goodness‐of‐fit test and graphical inspection of calibration plots comparing observed and predicted probabilities.

Performance metrics for both scales (sensitivity, specificity, positive predictive value, and negative predictive value) were calculated using cutoff scores optimized by the Youden index. The Youden index identified ≤ 10 as the optimal Braden cutoff and ≥ 15 as the optimal COMHON cutoff in this sample. For reference, the original published COMHON high‐risk threshold (≥ 14) is also reported in Table 1. Confidence intervals for all metrics are calculated using the Clopper–Pearson exact method and reported in Table 1. Meta‐analytic evidence supporting the evaluation of Braden Scale performance in critical care populations was additionally considered [16].

All analyses were conducted using IBM SPSS Statistics, Version 24 (IBM Corp., Armonk, NY, USA). Statistical significance was defined as a two‐sided *p* value < 0.05.

### 2.8. Ethics Statement

The study was conducted in accordance with the principles of the Declaration of Helsinki and its subsequent revisions. Ethical approval was obtained from the Research Ethics Committee of Fundación Jiménez Díaz (CEIm‐FJD), Madrid, Spain (code EO177‐23_HUQM), following evaluations held on 27 June 2023 and 26 September 2023. Given the retrospective, observational, and noninterventional design of the study, the requirement for informed consent was waived by the ethics committee. All data were obtained from routinely collected clinical records and were fully anonymized prior to analysis, ensuring confidentiality and compliance with applicable data protection regulations.

## 3. Results

Table 2 presents the baseline characteristics of the study population. A total of 476 patients admitted to the ICU wereincluded, with a mean age of 63.7 years (SD: 14.6) and a predominance of male patients (58.2%). Clinical severity at admission was high, as reflected by a mean SAPS‐3 score of 51.1 points (SD: 17.8). Diabetes mellitus was present in 23.1% of the patients, and cardiovascular, respiratory, and gynecological conditions were the most frequent admission diagnoses. Of the 476 patients included, 9 (1.9%) developed an ICU‐acquired pressure injury within the first 24 h after ICU admission. A STROBE‐compliant participant flow diagram is presented in Figure 1.

**TABLE 2:** Baseline characteristics of the study population, organized by domain.

Variable	*n*	Value
Demographic and clinical characteristics		
Age (years)	473	63.7 ± 14.6
Height (cm)	391^a^	172.0 ± 8.7
Weight (kg)	475	79.0 ± 15.9
SAPS‐3 score	456	51.1 ± 17.8
Male sex	474	276 (58.2%)
Diabetes mellitus	467	108 (23.1%)
Primary diagnosis category		
Cardiological		122 (25.6%)
Respiratory		100 (21.0%)
Gynecological		85 (17.9%)
Digestive		55 (11.6%)
Neurological		50 (10.5%)
Oncological		39 (8.2%)
Urological		11 (2.3%)
Nephrological		7 (1.5%)
Maxillofacial		5 (1.1%)
Hematological		2 (0.4%)
Preventive measures and outcomes		
Pressure‐redistributing mattress (yes)		130 (27.3%)
ICU‐acquired pressure injury at 24 h (yes)		9 (1.9%)
Braden Scale: subscale scores		
Sensory perception	471	2.64 ± 1.24
Moisture exposure	471	3.09 ± 0.91
Activity	471	1.28 ± 0.56
Mobility	471	2.11 ± 0.91
Nutrition	471	2.52 ± 0.75
Friction/shear	469	1.67 ± 0.67
Braden total score	476	13.3 ± 3.48
COMHON Index: domain scores		
Level of consciousness	473	2.08 ± 1.33
Mobility	473	2.57 ± 0.70
Hemodynamic status	473	1.87 ± 1.02
Oxygenation	473	2.54 ± 1.43
Nutrition	473	2.34 ± 1.00
COMHON total score	476	11.4 ± 3.79

*Note:* Values are presented as mean ± standard deviation for continuous variables and absolute frequency (%) for categorical variables. Overall *N* = 476 unless otherwise indicated. SAPS‐3: Simplified Acute Physiology Score III; COMHON: Consciousness, Mobility, Hemodynamics, Oxygenation, Nutrition.

^a^Height was missing in 17.8% of patients (*n* = 85); variable‐specific *n* values are indicated for variables with missing data only.

**FIGURE 1:**
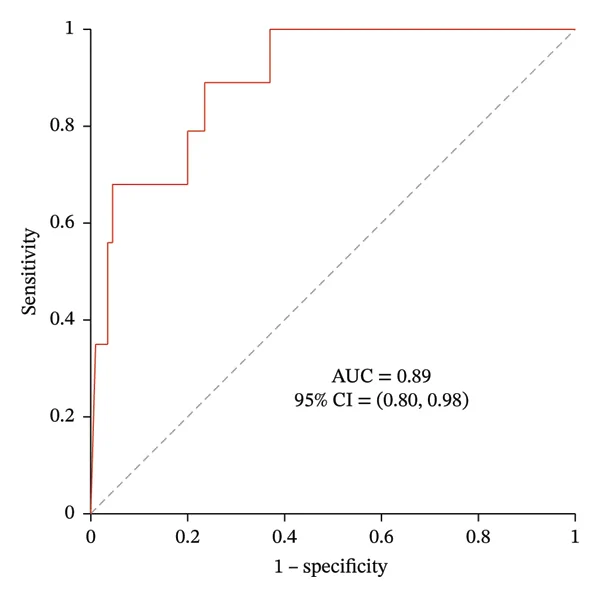
Receiver operating characteristic (ROC) curve of the final multivariable predictive model (Table 4) (AUC = 0.89; 95% CI: 0.80–0.98). This curve corresponds to the combined multivariable model incorporating subscales from both instruments, not to either scale individually. Individual AUC values for the Braden Scale total score and the COMHON Index total score are reported in the Results section. The optimal threshold point identified by the Youden index is marked on the curve. Calibration: Hosmer–Lemeshow test *p* = 0.74.

The univariable analysis is presented in Table 3. Overall disease severity was directly associated with pressure injury occurrence (SAPS‐3: OR: 1.04; 95% CI: 1.01–1.09). Within the Braden Scale, higher sensory perception (OR: 0.23; 95% CI: 0.03–0.58), lower moisture exposure (OR: 0.35; 95% CI: 0.17–0.67), reduced friction or shear (OR: 0.19; 95% CI: 0.02–0.76), and a higher total Braden score (OR: 0.62; 95% CI: 0.44–0.82) were associated with a significantly lower risk of pressure injury. In contrast, COMHON domains reflecting physiological instability were associated with increased risk, including impaired level of consciousness (OR: 2.37; 95% CI: 1.38–5.24), reduced mobility (OR: 3.51; 95% CI: 1.24–10.7), hemodynamic instability (OR: 3.51; 95% CI: 1.58–11.9), and altered oxygenation (OR: 1.90; 95% CI: 1.11–4.31), as well as the total COMHON score (OR: 1.53; 95% CI: 1.20–2.14). The Braden Activity subscale showed no variability in this cohort, as all patients scored 1, consistent with the bedrest status characteristic of ICU admissions, and the OR could not be estimated (NC). This floor effect indicates that this domain contributes no discriminative information in the present population. The COMHON Nutrition subscale did not reach statistical significance (OR: 1.38; 95% CI: 0.74–2.52; *p* = 0.303). For Braden Scale subscales, higher scores indicate lower risk (protective direction); for COMHON Index subscales, higher scores reflect greater physiological compromise (risk direction). The direction of OR interpretation differs accordingly between instruments.

**TABLE 3:** Distribution of study variables according to pressure injury status and univariable logistic regression analyses.

Variable	No pressure injury (*n* = 467)	Pressure injury (*n* = 9)	OR (95% CI)	*p* value
Clinical severity				
SAPS‐3 score	51 (35.5–60)	62 (54–78)	1.04 (1.01–1.09)	0.016
Braden Scale subscales (higher score = lower risk)				
Sensory perception	3 (1–4)	1 (1–1)	0.23 (0.03–0.58)	< 0.001
Moisture exposure	3 (3–4)	2 (1–3)	0.35 (0.17–0.67)	0.002
Activity	1 (1–1)	1 (1–1)	NC (floor effect)	—
Mobility	2 (1–3)	2 (1.7–2)	0.65 (0.26–1.40)	0.276
Nutrition	3 (2–3)	2 (1.7–3)	0.50 (0.20–1.23)	0.129
Friction/shear	2 (1–2)	1 (1–1)	0.19 (0.02–0.76)	0.015
Braden total score	13 (11–16)	9 (9–10)	0.62 (0.44–0.82)	< 0.001
COMHON Index domains (higher score = greater physiological compromise)				
Level of consciousness	1 (1–4)	4 (3–4)	2.37 (1.38–5.24)	0.001
Mobility	3 (2–3)	3 (3–3)	3.51 (1.24–10.7)	0.017
Hemodynamic status	1 (1–3)	3 (3–3)	3.51 (1.58–11.9)	0.001
Oxygenation	3 (1–4)	4 (4–4)	1.90 (1.11–4.31)	0.017
Nutrition	2 (2–3)	2 (2–4)	1.38 (0.74–2.52)	0.303
COMHON total score	11 (8–15)	16 (16–18)	1.53 (1.20–2.14)	< 0.001
Demographic and clinical characteristics				
Age, years	65.5 (58–72)	70 (68–70)	1.00 (0.96–1.05)	0.952
Height, cm	172 (165–180)	165 (164–170)	0.95 (0.87–1.02)	0.163
Weight, kg	80 (70–86)	85 (80–85)	0.99 (0.95–1.04)	0.764
Male sex (ref.)	273 (98.9%)	3 (1.1%)	Reference	—
Female sex	192 (97.0%)	6 (3.0%)	2.64 (0.74–11.2)	0.136
Diabetes mellitus (yes)	352 (98.1%)	7 (1.9%)	1.10 (0.20–4.20)	0.895
Gynecological diagnosis	80 (94.1%)	5 (5.9%)	4.53 (0.88–44.8)	0.072
Preventive measures				
Pressure‐redistributing mattress (yes)	124 (95.4%)	6 (4.6%)	5.12 (1.43–21.8)	0.012

*Note:* Data are presented as median (IQR) for continuous variables and as *n* (%) for categorical variables. ORs with 95% CIs are estimated using univariable Firth’s penalized logistic regression. NC: not calculable (floor effect; all patients scored 1 on Activity subscale). For Braden Scale subscales, higher scores indicate lower risk; for COMHON Index subscales, higher scores reflect greater physiological compromise. Only diagnostic categories with at least one event are shown. SAPS‐3: Simplified Acute Physiology Score III. *p* < 0.05 is considered statistically significant.

Table 4 presents the multivariable analysis using Firth’s penalized logistic regression. In the final model, higher sensory perception (OR: 0.19; *p* = 0.014) and lower moisture exposure (OR: 0.37; *p* = 0.018) remained independently associated with a reduced risk of pressure injury. Impaired nutritional status as assessed by the COMHON Index was independently associated with higher odds (OR: 2.85; *p* = 0.014). Hemodynamic instability showed a clinically relevant effect size but did not reach statistical significance (OR: 1.91; *p* = 0.116). Notably, the final model incorporated subscales from both instruments, two from the Braden Scale and one from the COMHON Index, indicating that optimal discrimination in this sample draws complementary information from both tools rather than from either alone.

**TABLE 4:** Final multivariable Firth’s penalized logistic regression model: independent predictors of pressure injury occurrence within the first 24 h after ICU admission.

Variable	Odds ratio (OR)	95% confidence interval	*p* value
Sensory perception (Braden)	0.19	0.05–0.72	0.014
Hemodynamic instability (COMHON)	1.91	0.85–4.28	0.116
Moisture exposure (Braden)	0.37	0.16–0.84	0.018
Nutritional status (COMHON)	2.85	1.24–6.57	0.014

*Note:* Only variables retained in the final model after AIC‐guided stepwise selection are presented. Variables not shown were not retained in the final model. ORs > 1 indicate increased risk; ORs < 1 indicate protective association. EPV = 2.25; results are exploratory.

Abbreviations: OR, odds ratio; CI, confidence interval.

Tables 5 and 6 summarize the associations between Braden and COMHON scale scores, clinical severity, and pressure injury occurrence. Higher SAPS‐3 scores were significantly associated with lower Braden total scores (*ρ* = −0.41, *p* < 0.001) and with reduced sensory perception, moisture exposure, and friction/shear (all *p* < 0.001). Conversely, SAPS‐3 was positively correlated with the COMHON total score (*ρ* = 0.37, *p* < 0.001) and with domains related to level of consciousness, hemodynamic status, and oxygenation (all *p* < 0.001). Patients who developed pressure injuries within the first 24 h had significantly lower Braden scores and higher COMHON scores than those without pressure injuries (both *p* < 0.001).

**TABLE 5:** Spearman’s rank correlation between Braden Scale and COMHON Index scores and SAPS‐3 score.

Scale/domain	Spearman’s rho with SAPS‐3
Braden total score	−0.41
Sensory perception	−0.40
Moisture exposure	−0.32
Friction/shear	−0.29
COMHON total score	0.37
Level of consciousness	0.43
Hemodynamic status	0.26
Oxygenation	0.42

*Note:* Only domains demonstrating statistical significance in the univariable analysis are presented. Rho: Spearman’s rank correlation coefficient; SAPS‐3: Simplified Acute Physiology Score III. The *p* value corresponding to all correlation coefficients is < 0.001.

**TABLE 6:** Comparison of Braden Scale and COMHON Index scores according to pressure injury occurrence within the first 24 h after ICU admission.

Scale/domain	Median (IQR) without PI	Median (IQR) with PI	*p* value
Braden total score	13 (11–16)	9 (9–10)	< 0.001
Sensory perception	3 (1–4)	1 (1–1)	*p* < 0.001
Moisture exposure	3 (3–4)	2 (1–3)	0.002
Friction/shear	2 (1–2)	1 (1–1)	0.015
COMHON total score	11 (8–15)	16 (16–18)	< 0.001
Level of consciousness	1 (1–4)	4 (3–4)	*p* < 0.001
Hemodynamic status	1 (1–3)	3 (3–3)	*p* < 0.001
Oxygenation	3 (1–4)	4 (4–4)	0.016

*Note:* Data are presented as median (interquartile range). Group comparisons are performed using the Mann–Whitney *U* test. IQR, interquartile range.

Abbreviation: PI, pressure injury.

Figure 2 presents the ROC curve of the multivariable model (AUC = 0.89; 95% CI: 0.80–0.98). It should be noted that this curve corresponds to the final combined predictive model rather than to either the Braden Scale or the COMHON Index individually. Individual AUC values for the Braden total score and the COMHON total score in this sample were 0.84 (95% CI: 0.72–0.96) and 0.87 (95% CI: 0.78–0.96), respectively, and were not statistically different from each other (DeLong test *p* = 0.48), indicating comparable discriminative capacity for both scales at the total score level. The combined multivariable model achieved the highest AUC by integrating subscales from both instruments. Model calibration was acceptable (Hosmer–Lemeshow *p* = 0.74).

**FIGURE 2:**
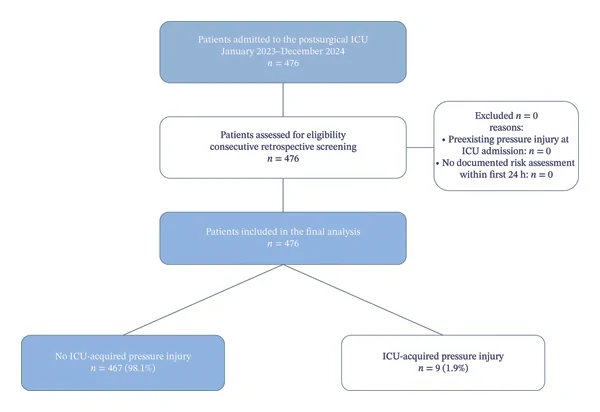
Participant flow diagram depicting patient identification, screening, and final inclusion for analysis. ICU: intensive care unit.

Table 1 presents the predictive performance metrics for both scales at the Youden‐optimized cutoff scores (Braden ≤ 10; COMHON ≥ 15). At these cutoffs, the Braden Scale demonstrated 100.0% sensitivity (95% CI: 66.4–100.0) and 77.1% specificity (95% CI: 73.0–80.8). The COMHON Index demonstrated 88.9% sensitivity (95% CI: 51.8–99.7) and 73.2% specificity (95% CI: 69.0–77.2). Both instruments showed very high negative predictive values (Braden: 100.0%, 95% CI: 99.0–100.0; COMHON: 99.7%, 95% CI: 98.4–100.0). The wide confidence intervals for sensitivity reflect the small number of events (*n* = 9) and should be interpreted with caution.

## 4. Discussion

In this study, clinically relevant differences were identified between the Braden Scale and the COMHON Index in the assessment of pressure injury risk in critically ill patients. Although both tools showed significant associations with the outcome, the results suggested differences in the clinical domains associated with pressure injury occurrence within the critical care context.

The association between higher SAPS‐3 scores and pressure injury occurrence supports the multifactorial nature of tissue damage in critically ill patients, where impaired perfusion, metabolic stress, and external mechanical load interact to reduce tissue tolerance. This interpretation is consistent with the conceptual framework proposed by Coleman et al. [1], which describes pressure injuries as consequences of inadequate perfusion, organ dysfunction, and metabolic stress, conditions frequently present in critically ill patients.

From the perspective of the Braden Scale, domains related to sensory perception, moisture exposure, and friction or shear demonstrated a significant protective effect. These findings converge with the original conceptual model described by Braden and Bergstrom [6] and its subsequent validation [5], confirming that the scale remains useful as a general screening tool and as a guide for basic nursing interventions. This convergence underscores the fundamental role of nursing care in pressure injury prevention.

Nevertheless, limitations of the Braden Scale when applied in ICUs have been reported. Previous studies have described reduced discriminative capacity in patients with deep sedation, mechanical ventilation, or vasoactive support, in whom several domains show limited variability [7‐8]. This observation is consistent with the present findings and suggests that, in populations with high clinical complexity, the Braden Scale tends to cluster patients into intermediate risk categories, potentially leading to an underestimation of true risk.

In contrast, the COMHON Index showed direct and consistent associations between its physiological domains and the development of pressure injuries. The COMHON Index was developed specifically for use in ICU populations [4] and has been validated in Australian [4] and Chinese Mandarin‐speaking cohorts [10], demonstrating acceptable reliability and convergent validity with the Braden Scale. The present study represents, to our knowledge, the first comparative evaluation of the two instruments in a Southern European ICU context. COMHON domains incorporate physiological parameters directly associated with tissue perfusion and oxygen delivery, which may explain their stronger association with pressure injury development in critically ill patients compared with the Braden Scale. This divergence is further supported by the biomechanical work of Gefen [2] and Linder‐Ganz et al. [17], which highlight the key role of perfusion and oxygenation in tissue integrity under sustained pressure.

The multivariable analysis reinforced the complementary, rather than mutually exclusive, nature of the two tools. After adjustment, sensory perception and moisture exposure, two Braden Scale subscales, remained independent protective factors, whereas impaired nutritional status, as assessed by the COMHON Index, emerged as an independent predictor of increased risk. This finding indicates that the optimal predictive model draws from both instruments: the Braden Scale captures functional and care‐related risk factors, while the COMHON Index contributes domain‐specific physiological information, particularly nutritional compromise. The results therefore support an integrative rather than substitutive interpretation of the two tools in ICU risk assessment. Although hemodynamic instability did not reach statistical significance after adjustment, the magnitude of its effect was consistent with previous research indicating its impact on capillary perfusion and tissue oxygenation.

The prespecified hypothesis of this study posited that the COMHON Index would demonstrate superior sensitivity compared with the Braden Scale. However, the observed results did not fully support this hypothesis: at the Youden‐optimized cutoff, the Braden Scale achieved higher sensitivity (100.0% vs. 88.9%). Furthermore, individual AUC values for both total scores were not statistically different, indicating comparable discriminative capacity at the total score level. These findings suggest that the findings do not establish that eitherinstrument is superior in this sample, and that the complementary use of subscales from both tools, as reflected in the final multivariable model, may represent the most clinically useful approach for risk stratification in this ICU population.

An important methodological consideration concerns the differential data collection approach for the two instruments: the Braden Scale was prospectively documented by nursing staff as part of routine clinical care, while COMHON scores were reconstructed retrospectively from clinical records. This asymmetry introduces a differential validity concern that potentially favors or disadvantages one instrument in ways that are difficult to quantify. Consequently, comparative performance metrics between the two instruments should be interpreted with caution, as observed differences may reflect operationalization differences rather than true instrument performance differences. The absence of inter‐rater reliability assessment for either instrument further limits the interpretation of the comparative performance metrics. Future prospective studies administering both instruments concurrently by trained nursing staff would be necessary to resolve this concern.

The use of pressure‐redistributing mattresses was significantly associated with pressure injury occurrence in univariable analysis (OR: 5.12; 95% CI: 1.43–21.8; *p* = 0.012). This paradoxical direction, mattress use associated with higher pressure injury risk, is consistent with confounding by indication: mattresses are prescribed reactively to patients already identified as high risk, so the observed association reflects nursing risk assessment practices rather than a causal harmful effect of the mattress. This variable was excluded from the multivariable model on this basis, following the methodological reasoning established for reactive interventions in observational studies. This finding does not imply that mattress use is harmful. The observed association is more likely attributable to patient case‐mix: patients who develop pressure injuries are also those most likely to have received a mattress precisely because of their high‐risk status. Residual confounding by unmeasured severity factors such as vasopressor dose, fluid balance, or duration of immobility may further explain the observed direction. These results underscore the inherent limitation of observational data when evaluating interventions that are systematically targeted to high‐risk individuals and support the need for prospective controlled evaluations of support surface allocation protocols in critically ill patients.

The discriminative capacity of the multivariable model was adequate for differentiating between patients with and without pressure injuries. In this context, the present results do not contradict the use of the Braden Scale but rather support its complementary role alongside setting‐specific tools such as the COMHON Index in ICUs.

From a clinical perspective, the incorporation of ICU‐specific tools such as the COMHON Index may facilitate earlier identification of patients requiring intensified preventive strategies, including repositioning protocols, advanced support surfaces, nutritional optimization, and closer hemodynamic monitoring. These findings may also support more individualized nursing decision‐making in critically ill populations characterized by high physiological instability.

From a prospective standpoint, the present findings provide a rationale for the design of multicenter studies to evaluate whether interventions targeting the optimization of oxygenation and hemodynamic stability, guided by the COMHON Index, are associated with a reduction in pressure injury occurrence. The COMHON Index bundle protocol, which recommends dynamic reassessment every 8 h to reflect changes in patient condition, may be particularly valuable in critically ill patients whose physiological status evolves rapidly over time.

### 4.1. Limitations of the Study

Several limitations of this study warrant acknowledgment. First, the retrospective design precludes causal inference. Second, the low event rate (*n* = 9; 1.9%) yielded an events‐per‐variable ratio of approximately 2.25, well below the recommended threshold of ≥ 10 [15]; although Firth’s penalized regression mitigates separation bias, model instability cannot be excluded and the multivariable results should be treated as exploratory. Third, residual confounding cannot be fully excluded given the observational nature of the study.

A fundamental methodological concern is the asymmetry between instruments: the Braden Scale was prospectively documented by nursing staff, while the COMHON Index was reconstructed retrospectively from clinical records. This operationalization bias may not accurately reflect real‐time bedside assessment, introduces a differential validity concern that cannot be quantified, and constrains the interpretation of comparative performance metrics. The absence of inter‐rater reliability assessment for either instrument further limits these comparisons.

Additionally, restricting the primary outcome to the first 24 h after ICU admission substantially limits the number of events and does not capture injuries developing over the full ICU stay; future studies should extend outcome assessment accordingly. The retrospective design precluded extension of the outcome window to subsequent ICU days in the present study; a prospective design with continuous event surveillance across the full ICU admission period is recommended to provide a more complete picture of pressure injury incidence and the temporal performance of both instruments. Finally, the single‐center design limits external generalizability to settings with different patient profiles or preventive protocols.

## 5. Conclusions

In conclusion, both the Braden Scale and the COMHON Index were associated with pressure injury occurrence in critically ill patients. The final multivariable model incorporated two Braden Scale subscales (sensory perception and moisture exposure) and one COMHON Index domain (nutritional status) as independent predictors, indicating that optimal discrimination in this sample draws complementary information from both instruments. Physiological domains of the COMHON Index, particularly nutritional status, provided incremental predictive information beyond Braden subscale scores. These findings should be interpreted cautiously given the retrospective single‐center design, the low event rate, and the methodological asymmetry between the two instruments. The multivariable results are exploratory and hypothesis‐generating rather than confirmatory. Further prospective multicenter studies are needed to confirm the clinical utility and generalizability of ICU‐specific risk assessment tools in pressure injury prevention. From a clinical standpoint, the dynamic nature of physiological deterioration in critically ill patients underscores the importance of repeated risk reassessment. Existing COMHON Index implementation frameworks recommend reassessment every 8 h, supporting a continuous monitoring approach aligned with the evolving clinical status of ICU patients.

## Author Contributions

Conceptualization, S.M.S. and A.R. Methodology, M.A.C.B. and J.O. Formal analysis, A.C.C., S.M.S., and M.V.C.T. Research, S.H.I., S.G.V., and M.V.C.T. Data interpretation, A.R. and M.G.P. Writing, M.A.C.B. and A.R. Revision and editing, M.A.C.B.

## Funding

This research did not receive any specific grants from public, commercial, or nonprofit funding agencies.

## Disclosure

All authors have read and approved the final version of the manuscript.

## Ethics Statement

The study was conducted in accordance with the principles of the Declaration of Helsinki and its subsequent revisions. Ethical approval was granted by the Research Ethics Committee of Fundación Jiménez Díaz (CEIm‐FJD), Madrid, Spain (code EO177‐23_HUQM), following evaluations held on 27 June 2023 and 26 September 2023.

## Consent

Informed consent was waived due to the retrospective and noninterventional nature of the study. All patient data were fully anonymized.

## Conflicts of Interest

The authors declare no conflicts of interest.

## Supporting Information

Additional supporting information can be found online in the Supporting Information section.

## Supporting information


**Supporting Information** STROBE Statement—Checklist of items that should be included in reports of cohort studies.

## Data Availability

The data that support the findings of this study are available from the corresponding author upon reasonable request.
